# Plasma Biomarker Concentrations Associated With Return to Sport Following Sport-Related Concussion in Collegiate Athletes—A Concussion Assessment, Research, and Education (CARE) Consortium Study

**DOI:** 10.1001/jamanetworkopen.2020.13191

**Published:** 2020-08-27

**Authors:** Cassandra L. Pattinson, Timothy B. Meier, Vivian A. Guedes, Chen Lai, Christina Devoto, Thaddeus Haight, Steven P. Broglio, Thomas McAllister, Christopher Giza, Daniel Huber, Jaroslaw Harezlak, Kenneth Cameron, Gerald McGinty, Jonathan Jackson, Kevin Guskiewicz, Jason Mihalik, Alison Brooks, Stefan Duma, Steven Rowson, Lindsay D. Nelson, Paul Pasquina, Michael McCrea, Jessica M. Gill

**Affiliations:** 1 National Institute of Nursing Research, National Institutes of Health, Bethesda, Maryland; 2 Institute for Social Science Research, University of Queensland, Brisbane; 3 Department of Neurosurgery, Medical College of Wisconsin, Milwaukee; 4 Center for Neuroscience and Regenerative Medicine, Uniformed Services University of the Health Sciences, Bethesda, Maryland; 5 The Henry M. Jackson Foundation for the Advancement of Military Medicine, Bethesda, Maryland; 6 Michigan Concussion Center, University of Michigan, Ann Arbor; 7 Department of Psychiatry, Indiana University School of Medicine, Indianapolis; 8 Department of Neurosurgery and Pediatrics, UCLA Steve Tisch BrainSPORT Program, University of California, Los Angeles; 9 Department of Epidemiology and Biostatistics, School of Public Health, Indiana University, Bloomington; 10 Keller Army Community Hospital, West Point, New York; 11 United States Air Force Academy, Colorado; 12 Matthew Gfeller Sport-Related Traumatic Brain Injury Research Center, Department of Exercise and Sport Science, University of North Carolina, Chapel Hill; 13 Department of Orthopedics and Sports Medicine, School of Medicine and Public Health, University of Wisconsin–Madison; 14 Department of Biomedical Engineering, Virginia Tech, Blacksburg; 15 Department of Physical Medicine and Rehabilitation, Uniformed Services University of the Health Sciences, Bethesda, Maryland

## Abstract

**Importance:**

Identifying plasma biomarkers associated with the amount of time an athlete may need before they return to sport (RTS) following a sport-related concussion (SRC) is important because it may help to improve the health and safety of athletes.

**Objective:**

To examine whether plasma biomarkers can differentiate collegiate athletes who RTS in less than 14 days or 14 days or more following SRC.

**Design, Setting, and Participants:**

This multicenter prospective diagnostic study, conducted by the National Collegiate Athletics Association–Department of Defense Concussion Assessment, Research, and Education Consortium, included 127 male and female athletes who had sustained an SRC while enrolled at 6 Concussion Assessment, Research, and Education Consortium Advanced Research Core sites as well as 2 partial–Advanced Research Core military service academies. Data were collected between February 2015 and May 2018. Athletes with SRC completed clinical testing and blood collection at preseason (baseline), postinjury (0-21 hours), 24 to 48 hours postinjury, time of symptom resolution, and 7 days after unrestricted RTS.

**Main Outcomes and Measures:**

A total of 3 plasma biomarkers (ie, total tau protein, glial fibrillary acidic protein [GFAP], and neurofilament light chain protein [Nf-L]) were measured using an ultrasensitive single molecule array technology and were included in the final analysis. RTS was examined between athletes who took less than 14 days vs those who took 14 days or more to RTS following SRC. Linear mixed models were used to identify significant interactions between period by RTS group. Area under the receiver operating characteristic curve analyses were conducted to examine whether these plasma biomarkers could discriminate between RTS groups.

**Results:**

The 127 participants had a mean (SD) age of 18.9 (1.3) years, and 97 (76.4%) were men; 65 (51.2%) took less than 14 days to RTS, and 62 (48.8%) took 14 days or more to RTS. Linear mixed models identified significant associations for both mean (SE) plasma total tau (24-48 hours postinjury, <14 days RTS vs ≥14 days RTS: −0.65 [0.12] pg/mL vs −0.14 [0.14] pg/mL; *P* = .008) and GFAP (postinjury, 14 days RTS vs ≥14 days RTS: 4.72 [0.12] pg/mL vs 4.39 [0.11] pg/mL; *P* = .04). Total tau at the time of symptom resolution had acceptable discrimination power (area under the receiver operating characteristic curve, 0.75; 95% CI, 0.63-0.86; *P* < .001). We also examined a combined plasma biomarker panel that incorporated Nf-L, GFAP, and total tau at each period to discriminate RTS groups. Although the analyses did reach significance at each time period when combined, results indicated that they were poor at distinguishing the groups (area under the receiver operating characteristic curve, <0.7).

**Conclusions and Relevance:**

The findings of this study suggest that measures of total tau and GFAP may identify athletes who will require more time to RTS. However, further research is needed to improve our ability to determine recovery following an SRC.

## Key Points

**Question:**

Are plasma biomarkers associated with a return-to-sport period of less than 14 days vs 14 days or more in male and female collegiate athletes following a sport-related concussion?

**Findings:**

This diagnostic study, which included 127 collegiate athletes who had sustained a sports-related concussion, found that higher total tau concentrations 24 to 48 hours after injury and at the time of symptom resolution as well as lower glial fibrillary acidic protein levels acutely postinjury were associated with return-to-sport decisions.

**Meaning:**

In this study, total tau and glial fibrillary acidic protein levels were associated with return to sport in male and female collegiate athletes following a sports-related concussion.

## Teaser

This diagnostic study examines whether plasma biomarkers can differentiate collegiate athletes who return to sport in less than 14 days vs 14 days or more following a sports-related concussion.

## Introduction

Sport-related concussion (SRC) is prevalent across multiple sports and can be challenging to manage clinically.^1^ Return-to-sport (RTS) decisions are essential for ensuring safety, given that the risk of subsequent concussion is increased if an athlete returns to play prior to full clinical and neurobiological recovery.^2,3^ Furthermore, early RTS may have negative consequences on long-term neurologic health. Findings suggest that athletes may have increased vulnerability to subsequent concussions, with subsequent increased risk of chronic symptoms, functional impairments, and possibly neurodegenerative disease.^2,4,5^ Peripheral blood-based biomarkers present an opportunity to objectively measure proteins that may be indicative of injury severity alongside neuronal recovery. Thus, following diagnosis of SRC, blood biomarkers may allow us to identify those athletes at risk of prolonged recovery, with the aim of ensuring athletes are not only asymptomatic but also show signs of neuronal recovery when returning to sport.

SRC has a complex pathophysiology comprising multiple neurobiological effects. Candidate biomarkers of these pathophysiologic mechanisms include total tau and neurofilament light chain protein (Nf-L), microtubule-associated proteins from neurons, and glial fibrillary acidic protein (GFAP), released from astrocytes. Recent research demonstrated that elevated total tau levels in blood 6 hours postconcussion were associated with prolonged RTS in collegiate athletes.^6^ These findings are consistent with data from professional ice hockey players, which found that elevations in total tau at 1 hour postinjury and Nf-L at 1, 12, 36, and 144 hours postinjury were associated with longer RTS time (ie, >10 days).^7,8^ However, not all studies support these findings, with a 2018 study reporting no significant associations between Nf-L or total tau with RTS.^9^ Furthermore, research that investigates other classic markers of mild traumatic brain injury (mTBI), such as GFAP and ubiquitin C-terminal hydrolase-L1 (UCH-L1), which have been linked to persistent symptoms and injury severity,^10^ is lacking in collegiate athlete populations following SRC.

The aim of this study was to assess the utility of plasma biomarkers, including total tau, Nf-L, GFAP, and UCHL-1, in discriminating RTS decisions among collegiate athletes who sustained an SRC. Current guidelines from the National Collegiate Athletics Association (NCAA) indicate that collegiate athletes will typically be asymptomatic within 14 days after an SRC.^11^ These statistics also aligned with the median (interquartile range [IQR]) RTS time for the Concussion Assessment, Research, and Education (CARE) Consortium sample (whole sample, 12.9 [8.7-20.7] days; men, 12.2 [8.3-19.7] days; women, 13.7 [9.1-23.6] days).^12^ Thus, 14 days was the cut point used to determine whether blood-based biomarkers could differentiate athletes with SRC who were able to RTS in less than 14 days compared with those who took 14 days or more to RTS.

## Methods

This was a multicenter, prospective diagnostic study led by the Advanced Research Core (ARC) of the NCAA–Department of Defense CARE Consortium. This study was approved by the Medical College of Wisconsin institutional review board and the Human Research Protection Office at the US Army Medical Research and Material Command. Written informed consent was obtained from all participants. The study followed the Standards for Reporting of Diagnostic Accuracy (STARD) reporting guideline.

### Participants

Of the CARE ARC and partial-ARC databases, 308 participants were identified as having sustained an SRC at some point during the study. Of these participants, 139 had provided a blood sample at postinjury. Of those, 12 (8.6%) had missing RTS information. Thus, the final sample included 127 participants who had sustained an SRC with both RTS information and a blood sample provided at postinjury.

### Procedures

The CARE Consortium ARC has been detailed elsewhere.^13^ Briefly, the protocol involves preseason baseline clinical testing and blood biospecimen collection in male and female athletes competing in contact sports, including football, soccer, lacrosse, ice hockey, and rugby, at 6 participating US universities, including 2 military service academies. Concussion (or mTBI) was defined according to the consensus definition from the Department of Defense evidence-based guidelines and could include observed or documented alterations of consciousness and/or mental state (within 24 hours), less than 30 minutes of loss of consciousness (LOC), and/or posttraumatic amnesia lasting for as long as 1 day.^14^ Alongside the preseason baseline testing, the CARE postinjury protocol included follow-up clinical testing and blood collection in athletes with concussion at 5 periods: 0 to 21 hours postinjury, 24 to 48 hours postinjury, time of symptom resolution (ie, athletes were asked to return for testing when they had been cleared to begin the RTS progression),^13^ 7 days after unrestricted RTS, and 6 months postinjury. Because the aim of this study was to investigate change in acute plasma biomarker concentrations following injury, the 6-month postinjury period was excluded from analyses.

### RTS

Current guidelines from the NCAA indicate that collegiate athletes will typically be asymptomatic within 2 weeks following an SRC.^11^ In line with these guidelines, the median (IQR) RTS time for the entire CARE sample was 12.9 (8.7-20.7) days.^12^ Furthermore, this point closely corresponded with the individual being asymptomatic, which was required for an RTS decision to be made. As such, we used a medium-split model to determine whether there were observable differences in peripheral blood biomarkers that could differentiate athletes with SRC who were able to RTS in less than 14 days compared with those who took more than 14 days to RTS.

### Blood Collection

Nonfasting blood samples were collected by venipuncture at preseason baseline and at all subsequent postinjury periods that the athletes attended. A 10-mL red-top tube for plasma was collected at each period. Tubes were centrifuged within 30 minutes of collection for 15 minutes at 1500 relative centrifugal force and then aliquoted. The cryovials were stored upright in a −80 °C freezer until shipped on dry ice to the CARE Consortium biorepository at Indiana University School of Medicine for long-term storage.

### Biomarker Analysis

Single molecular array technology (SIMOA, Quanterix Corp), located at the National Institutes of Health, was used to measure the level of all biomarkers. The use of SIMOA for protein detection maximizes sensitivity, with detection ability between 100 to 1000 times that of enzyme-linked immunosorbent assay methods.^15^ Multiplex technology was used to simultaneously quantify UCH-L1, total tau, Nf-L, and GFAP. Assays were batched to minimize variability, with each batch run using published standard operating procedures to ensure reliability. Groups were distributed randomly across plates, and longitudinal samples from the same individual were run on the same plate to reduce potential batch effects. All samples were analyzed in duplicate. In the rare instances in which coefficients of variance (CV) exceeded 20%, samples were rerun. If either intra-assay or interassay CVs were greater than 20% for either measure, data were not used. The mean CVs for each plasma protein were the following for data included in the analyses: total tau, 7.92%; Nf-L, 4.59%; and GFAP, 3.07%. The limits of detection are as follows: total tau, 0.0146 pg/mL; Nf-L, 0.038 pg/mL; GFAP, 0.211 pg/mL; and UCH-L1, 1.05 pg/mL. For UCH-L1, approximately 42% of samples were either below the detection limit or had CVs above 20%; therefore, UCH-L1 was not reported.

### Statistical Analysis

Statistical analyses were performed using SPSS statistical software version 25.0 (IBM Corp). Statistically significant results were declared at the nominal significance level α = .05. Time from injury to blood draw was calculated to ensure that the draw occurred within the prescribed protocol window.

The postinjury period had the most missing data owing to late reporting or difficulties in data collection (eg, away games). However, additional analysis of those with and without missing biomarker data at the postinjury period found that the groups did not significantly differ on any demographic variables (eTable in the Supplement).

Group comparisons of demographic characteristics and clinical outcomes were evaluated using independent-sample *t* tests for continuous variables and Fisher exact tests for categorical variables. Biomarker levels were natural log-transformed owing to the skewness of their distributions. Linear mixed models were used to evaluate changes in biomarker levels within athletes over time as a function of RTS group, with visit period modeled as a repeated factor (ie, postinjury, 24-48 hours postinjury, time of symptom resolution, and 7 days after unrestricted RTS), RTS group (ie, <14 days and ≥14 days), the group × period interaction, and participant as random factor. All hypotheses were 2-sided, with Bonferroni adjustments applied to all post hoc pairwise comparisons.

Receiver operating characteristic curves and area under the curve (AUC) with 95% CIs were used to quantify the ability of significant biomarkers identified to discriminate between athletes with less than 14 days RTS and those with 14 days or more before RTS. Finally, we wanted to examine whether a combination of biomarkers at each period could discriminate RTS duration. Therefore, we combined all 3 plasma biomarker concentrations at each period using binomial logistic regression models.

## Results

Table 1 summarizes the sample characteristics for the athletes with RTS less than 14 days postinjury and those with RTS 14 days or later. Participants in this study were aged 17 to 23 years (mean [SD] age, 18.9 [1.3] years); most were men (97 [76.4%]) and White individuals (82 [64.6%]). Of the 127 athletes, the median split in RTS was at 14 days postinjury. That is, 65 (51.1%) had RTS less than 14 days and 62 (48.8%) had RTS of 14 days or more. The groups did not differ on any of the demographic variables of age, sex, race, anthropometric data, or number of prior concussions. Most athletes had no prior concussions (71 [55.9%]). The group that took less than 14 days before RTS had significantly higher incidence of LOC compared with the group that took 14 days or more before RTS (7 [10.8%] vs 0; *P* = .01) and a longer mean (SD) time participating in sport (11.3 [4.4] years vs 8.8 [3.4] years; *P* = .004). Furthermore, baseline concentrations of natural log-transformed total tau, GFAP, and NF-L levels did not significantly differ between the groups. Table 1 presents sample characteristics for the 2 RTS groups.

**Table 1.** Demographic and Clinical Characteristics of Athletes in Each RTS Group

Characteristic	No. (%) by RTS group		*P* value
	<14-d (n = 65)	≥14-d (n = 62)	
Age, mean (SD), y	19.1 (1.4)	18.7 (1.2)	.19
Men	51 (78.5)	46 (74.2)	.57
Height, mean (SD), cm	179.6 (9.7)	178.6 (12.2)	.57
Weight, mean (SD), kg	84.3 (19.2)	82.9 (21.5)	.71
Duration of sport participation, mean (SD), y	11.31 (4.4)	8.8 (3.4)	.004
Race			
White	43 (66.2)	39 (62.9)	.55
African American	12 (18.5)	12 (19.4)	
Asian	3 (4.6)	5 (8.1)	
Hawaiian or Pacific Islander	0	2 (3.2)	
Multiple	6 (9.2)	4 (6.5)	
Unknown or not reported	1 (1.5)	0	
Ethnicity			
Non-Hispanic	54 (83.1)	55 (88.7)	.63
Hispanic	4 (6.2)	2 (3.2)	
Unknown or not reported	7 (10.8)	5 (8.1)	
With ADHD	6 (9.2)	1 (1.6)	.12^a^
Sport			
Football	24 (43.6)	22 (53.7)	.11
Ice hockey	4 (7.3)	0	
Lacrosse	3 (5.5)	3 (7.3)	
Rugby	5 (9.1)	3 (7.3)	
Soccer	14 (25.5)	5 (12.2)	
Other	5 (9.1)	8 (17.0)	
PTA	12 (18.5)	12 (19.4)	.90
LOC, yes	7 (10.8)	0	.01^a^
No. of prior concussions			
0	32 (50.0)	39 (63.9)	.13
1	23 (35.9)	20 (32.8)	
2	7 (10.9)	2 (3.3)	
≥3	2 (3.1)	0	
Time to symptom resolution, median (IQR), d	4.0 (3.4)	11.9 (6.9)	<.001^b^
Baseline biomarker concentrations, mean (SD), LN pg/mL			
Total tau	−0.2 (0.6)	−0.2 (0.8)	.85
GFAP	4.1 (0.4)	4.0 (0.4)	.43
Nf-L	1.8 (0.5)	1.6 (0.5)	.06

Abbreviations: ADHD, attention-deficit/hyperactivity disorder; GFAP, glial fibrillary acidic protein; IQR, interquartile range; LN, natural log-transformation; LOC, loss of consciousness; Nf-L, neurofilament light chain protein; PTA, posttraumatic amnesia; RTS, return to sport.

^a^ Result from 2-tailed Fisher exact test.

^b^ Result from nonparametric Mann-Whitney *U* test.

### RTS Biomarkers

Linear mixed models identified significant associations for both mean (SE) plasma total tau (24-48 hours postinjury, <14 days RTS vs ≥14 days RTS: −0.65 [0.12] pg/mL vs −0.14 [0.14] pg/mL; *P* = .008) and GFAP (postinjury, 14 days RTS vs ≥14 days RTS: 4.72 [0.12] pg/mL vs 4.39 [0.11] pg/mL; *P* = .04). For total tau, there was a significant interaction between period and RTS group (*F*_2.4, 128.2_ = 3.66; *P* = .02; *n_p_^2^* = 0.065). Bonferroni-adjusted pairwise post hoc comparisons revealed that at both 24 to 48 hours postinjury and at the time of symptom resolution, the group that took 14 days or more to RTS had higher total tau concentrations than the group that took less than 14 days to RTS (Table 2). For GFAP, there was a significant interaction between period and RTS groups (*F*_1.5, 117.8_ = 3.48; *P* = .04; *n_p_^2^* = 0.043). Pairwise post hoc comparisons indicated that the group that took 14 days or more to RTS had significantly lower concentrations of GFAP than the group that took less than 14 days at the postinjury period (Table 2). There was also a significant interaction between period and RTS groups (*F*_1.9, 144.0_ = 3.34; *P* = .04; *n_p_^2^* = 0.042) for Nf-L. However, pairwise post hoc comparisons revealed no significant associations after Bonferroni adjustment (Table 2).

**Table 2.** Pairwise Post Hoc Comparisons of Biomarker Concentrations Between Athletes Who Had Sustained a Sport-Related Concussion, by RTS Group^a^

Biomarker	Level, mean (SE), by RTS group, pg/mL		*P* value	Mean difference (95% CI)
	<14 d	≥14 d		
Total tau				
Participants, No.	31	24	NA	NA
Postinjury	−0.06 (0.13)	0.24 (0.14)	.12	−0.29 (−0.68 to 0.08)
24-48 h postinjury	−0.65 (0.12)	−0.14 (0.14)	.008	−0.51 (−0.88 to −0.14)
Time of symptom resolution	−0.54 (0.12)	0.17 (0.14)	<.001	−0.71 (−1.09 to −0.34)
7 d after unrestricted RTS	−0.30 (0.11)	−0.13 (0.12)	.31	−0.17 (−0.50 to 0.16)
GFAP				
Participants, No.	37	42	NA	NA
Postinjury	4.72 (0.12)	4.39 (0.11)	.04	0.33 (0.01 to 0.65)
24-48 h postinjury	4.37 (0.09)	4.28 (0.08)	.45	0.09 (−0.15 to 0.32)
Time of symptom resolution	4.26 (0.06)	4.21 (0.06)	.59	0.04 (−0.12 to 0.21)
7 d after unrestricted RTS	4.27 (0.07)	4.16 (0.06)	.23	0.11 (−0.07 to 0.29)
Nf-L				
Participants, No.	36	42	NA	NA
Postinjury	1.97 (0.07)	1.92 (0.07)	.59	0.05 (−0.14 to 0.25)
24-48 h postinjury	1.89 (0.07)	1.87 (0.06)	.81	0.02 (−0.16 to 0.21)
Time of symptom resolution	1.98 (0.08)	1.83 (0.07)	.17	0.15 (−0.07 to 0.37)
7 d after unrestricted RTS	2.05 (0.09)	1.85 (0.08)	.09	0.20 (−0.03 to 0.44)

Abbreviations: GFAP, glial fibrillary acidic protein; NA, not applicable; Nf-L, neurofilament light chain protein; RTS, return to sport.

^a^ All variables have been natural log–transformed and the *P* values and 95% CIs of the difference have been adjusted for multiple comparisons using Bonferroni adjustment.

We then conducted receiver operating characteristic curve analysis to examine whether the biomarkers and periods identified as significant could discriminate between athletes with 14 days or more before RTS from those with less than 14 days before RTS. The analysis indicated that total tau at the period of symptom resolution had acceptable^16^ discrimination power (AUC, 0.75; 95% CI, 0.63-0.86; *P* < .001) (Table 3).

**Table 3.** Receiver Operating Characteristic Curve Results

Variable^a^	AUC (SE) [95% CI]	*P* value	Cut point	Sensitivity	Specificity	PPV	NPV
Tau							
24-48 h postinjury	0.66 (0.06) [0.53-0.78]	.02	−0.72	0.82	0.49	0.65	0.72
Time of symptom resolution	0.75 (0.06) [0.63-0.86]	<.001	−0.41	0.79	0.51	0.63	0.68
GFAP, postinjury	0.40 (0.07) [0.27-0.54]	.15	4.26	0.66	0.63	0.53	0.51

Abbreviations: AUC, area under the receiver operating characteristic curve; GFAP, glial fibrillary acidic protein; NPV, negative predictive value; PPV, positive predictive value.

^a^ All variables have been natural log–transformed.

We also examined how well a combined biomarker panel, which incorporated Nf-L, GFAP, and total tau at each period, discriminated between athletes with 14 days or more before RTS vs those with less than 14 days before RTS. At postinjury, no single biomarker significantly discriminated between the groups when examined individually. However, when the biomarkers were combined, they reached significance, but were poor^16^ at distinguishing the groups (AUC, 0.63; 95% CI, 0.53-0.73; *P* = .02). At 24 to 48 hours postinjury, total tau individually was significant but had a low AUC (AUC, 0.65; 95% CI, 0.53-0.78; *P* = .02). The combination of all 3 plasma biomarkers added little to the prognostic ability of total tau individually at the 24 to 48 hours postinjury period (AUC, 0.67; 95% CI, 0.55-0.79; *P* = .01). A similar pattern was observed at the period of symptom resolution, with total tau once again being significant and able to discriminate between the RTS groups. The combination of biomarkers was also significant but added little to the prognostic ability of the model (AUC, 0.67; 95% CI, 0.57-0.77; *P* = .002).

## Discussion

Clinical management of SRC is challenging and further complicated by the lack of objective prognostic indicators to help inform clinical decisions, particularly for athletes who may be at risk of prolonged recovery. In this study, we examined the utility of blood biomarkers that may be associated with RTS decisions in 127 NCAA male and female student-athletes who sustained an SRC during sport participation. We found that athletes with less than 14 days before RTS had significantly higher total tau at the 24 to 48 hour period and at time of symptom resolution as well as significantly lower GFAP postinjury. Total tau was fair at discriminating those with 14 days or more before RTS vs those with less than 14 days before RTS, especially at the time of symptom resolution. Contrary to prior studies,^7,8^ Nf-L was not significantly associated with RTS decisions.

Our results indicate that athletes with 14 days or more before RTS had higher total tau at the 24 to 48 hour period, which remained elevated even when players reported being clinically asymptomatic. This result is consistent with prior studies, which have found that higher concentrations of total tau are associated with a longer RTS time after SRC in collegiate and professional athletes.^6,8^ While the 2 RTS groups in the current study did not differ significantly on the number of prior concussions sustained, more research is needed to identify whether there are any long-term effects of these short-term spikes in total tau that might alter the regulation mechanisms of tau; eg, the glymphatic system^17^ or subsequent vulnerability to dysregulation of tau and hyperphosphorylation.^18,19^

In contrast to the tau findings, levels of GFAP at the postinjury period were lower in athletes with SRC who took 14 days or more before RTS compared with those who took less than 14 days before RTS. These results are counterintuitive, given that they are in contrast with prior studies in civilian patients with mTBI. A possible explanation for the difference in our findings may be that civilian mTBI studies often include patients with a more severe gradient of injury, even within the mild classification, such as patients with evidence of structural brain injury on head computed tomography. For example, higher GFAP levels acutely following an mTBI are associated with having lesions detected by both computed tomography and magnetic resonance imaging^20^ and by identifying athletes with an SRC compared with controls with no injuries.^21^ Indeed, our group has previously reported that higher GFAP concentrations were associated with SRCs with LOC.^22^ Therefore, these results may actually support the association, given that the group with less than 14 days before RTS did report significantly higher incidence of LOC (Table 1). Alternatively, it may be that any increase in GFAP following the injury, potentially after the 48-hour sampling frame, may have been missed due to the timing of the blood samples.^23,24^ Whatever the cause of this finding, it is evident that research to better understand the release kinetics and significance of perturbations to the normally expected temporal profile of GFAP is warranted, particularly if this marker is to be considered as a guide to treatment options related to SRC.

Nf-L levels did not discriminate RTS duration in this cohort. Elevated levels of Nf-L are believed to reflect axonal damage due to injury or degeneration, which is a possible determinant of outcomes following concussions.^25,26,27^ Our findings expand on a previous study^9^ that reported no significant difference in plasma Nf-L levels at 6 and 14 days post-SRC relative to their baseline concentrations in a cohort of US football and hockey college athletes. However, prior studies have found that plasma Nf-L concentrations at 1, 12, 36, and 144 hours after SRC could discriminate players with more than 10 days before RTS from those with 10 days or less before RTS in professional hockey players.^8^ Disparities in these findings might result from cohort differences in age and previous concussion history. College-aged cohorts tend to be younger than professional athletes, which might result in differential responses to trauma. Moreover, professional players may have been exposed to a larger number of concussions and repetitive head impact exposures during the course of the season or their careers compared with college athletes. Accordingly, Nf-L has been linked to repetitive concussive impacts in contact-sport athletes.^7,25,28^ Indeed, our laboratory has previously observed that plasma Nf-L concentrations were elevated in military personnel who had sustained repetitive TBIs compared with those with 1 or 2 TBIs, but no differences were observed between control and TBI groups.^29^ Despite recent progress in SRCs, the prognostic value of Nf-L is yet to be fully ascertained. We hypothesize that Nf-L has limited sensitivity in milder cases of SRC and may become more evident as the number of SRCs sustained increases. Indeed, in this cohort, most participants (55.9%) reported having no prior concussions. Future studies should further evaluate the potential of Nf-L in mTBI patients, and it may be worthwhile to monitor Nf-L longitudinally across athletic careers.

### Limitations

Although this article uses data from, to our knowledge, the largest prospective sample of SRC in athletes to date, there are a number of limitations to note. Specifically, from the larger CARE Consortium pool of participants, our sample included a relatively small number of athletes who sustained an SRC with complete data on RTS and blood biomarkers. As such, the findings may not generalize to the wider collegiate community. However, the CARE Consortium study is ongoing, and we anticipate that we will be able to continue to gather a more representative sample to replicate and extend this work. Furthermore, because of the nature of the injury itself (occurring at any time of day), alongside participant schedules, we were unable to standardize the time of biomarker collection at each of the periods. Therefore, we were unable to rule out the influence of circadian timing on variations in biomarkers. There is no research to suggest that plasma concentrations of total tau, GFAP, or Nf-L show a diurnal pattern in humans; however, a relationship between sleep-wake disturbances and tau accumulation has been reported.^30,31^ Future studies, which account for sleep-wake patterns pre-injury and postinjury as well as before blood sampling present an intriguing and growing line of investigation. We were also unable to determine whether the changes in the biomarkers observed were owing to central or peripheral mechanisms. Future work will determine the relationship between the selected biomarkers and more direct measures of neuropathology (ie, neuroimaging). RTS is an outcome affected by several extraneous variables, including symptom recovery time and clinical management practices at each institution. In line with this, it was noted that the group that took less than 14 days to RTS had significantly more years of sports participation than those who took 14 or more days to RTS. One rationale for this difference is that more years of sports participation results in players having a better understanding of when they are fit to RTS. However, the number of prior concussions did not differ. As such, it may also be that players with more years of sporting participation feel more compelled or more pressure to RTS earlier. Further research into the individual psychological determinants of RTS may be needed to elucidate these findings further. This investigation is a first step in the process of identifying biomarkers that are associated with duration of recovery; however, it is evident that the need for continued longitudinal tracking of these athletes, including blood biomarkers alongside clinical outcomes, remains.

## Conclusions

Biomarkers that are associated with duration of recovery after SRC are needed to improve the clinical management of concussion. In this study, higher total tau concentrations at 24 to 48 hours postinjury and at the time of symptom resolution and lower GFAP concentrations at the postinjury period were associated with protracted RTS (ie, ≥14 days) in male and female collegiate athletes following SRC. Although preliminary, the current results highlight the potential role of biomarkers in tracking neuronal recovery, which may be associated with duration of RTS. Continued longitudinal tracking of this cohort is underway. We will continue to explore this association between biomarkers and their ability to track recovery in male and female athletes following SRC.

## Notes

Supplement. **eTable.** Demographic Characteristics of Participants With and Without Missing Biomarker Data at the Postinjury Period

## References

[1] Langlois JA, Rutland-Brown W, Wald MM The epidemiology and impact of traumatic brain injury: a brief overview. J Head Trauma Rehabil. 2006;21(5):375-378. doi:10.1097/00001199-200609000-00001 16983222

[2] McKee AC, Cantu RC, Nowinski CJ, Chronic traumatic encephalopathy in athletes: progressive tauopathy after repetitive head injury. J Neuropathol Exp Neurol. 2009;68(7):709-735. doi:10.1097/NEN.0b013e3181a9d503 19535999 PMC2945234

[3] McCrea M, Broglio S, McAllister T, ; CARE Consortium Investigators Return to play and risk of repeat concussion in collegiate football players: comparative analysis from the NCAA Concussion Study (1999-2001) and CARE Consortium (2014-2017). Br J Sports Med. 2020;54(2):102-109. doi:10.1136/bjsports-2019-100579 31036562

[4] Manley G, Gardner AJ, Schneider KJ, A systematic review of potential long-term effects of sport-related concussion. Br J Sports Med. 2017;51(12):969-977. doi:10.1136/bjsports-2017-097791 28455362 PMC5466926

[5] Slobounov S, Slobounov E, Sebastianelli W, Cao C, Newell K Differential rate of recovery in athletes after first and second concussion episodes. Neurosurgery. 2007;61(2):338-344. doi:10.1227/01.NEU.0000280001.03578.FF 17762746

[6] Gill J, Merchant-Borna K, Jeromin A, Livingston W, Bazarian J Acute plasma tau relates to prolonged return to play after concussion. Neurology. 2017;88(6):595-602. doi:10.1212/WNL.0000000000003587 28062722 PMC5304458

[7] Shahim P, Tegner Y, Wilson DH, Blood biomarkers for brain injury in concussed professional ice hockey players. JAMA Neurol. 2014;71(6):684-692. doi:10.1001/jamaneurol.2014.367 24627036

[8] Shahim P, Tegner Y, Marklund N, Blennow K, Zetterberg H Neurofilament light and tau as blood biomarkers for sports-related concussion. Neurology. 2018;90(20):e1780-e1788. doi:10.1212/WNL.0000000000005518 29653990 PMC5957307

[9] Wallace C, Zetterberg H, Blennow K, van Donkelaar P No change in plasma tau and serum neurofilament light concentrations in adolescent athletes following sport-related concussion. PLoS One. 2018;13(10):e0206466. doi:10.1371/journal.pone.0206466 30372457 PMC6205645

[10] Shahim P, Tegner Y, Marklund N, Astroglial activation and altered amyloid metabolism in human repetitive concussion. Neurology. 2017;88(15):1400-1407. doi:10.1212/WNL.0000000000003816 28283595 PMC5386435

[11] NCAA Interassociation consensus: diagnosis and management of sport-related concussion best practices. Accessed July 19, 2019. http://www.ncaa.org/sites/default/files/SSI_ConcussionBestPractices_20170616.pdf

[12] McAllister TW, Broglio SP, McCrea MA, Evolution and findings of the CARE Consortium: a pivot to studying cumulative and persistent effects of concussion and repetitive head impact exposure. Paper presented at: Military Health System Research Symposium; August 20-23, 2018; Kissimmee, Florida. Accessed July 28, 2020. https://docplayer.net/98038631-Mhsrs-symposium-guide-august-20-23-2018-gaylord-palms-resort-convention-center-kissimmee-fl-military-health-system-research-symposium.html

[13] Broglio SP, McCrea M, McAllister T, ; CARE Consortium Investigators A national study on the effects of concussion in collegiate athletes and us military service academy members: the NCAA-DoD Concussion Assessment, Research and Education (CARE) Consortium structure and methods. Sports Med. 2017;47(7):1437-1451. doi:10.1007/s40279-017-0707-1 28281095 PMC5488134

[14] Carney N, Ghajar J, Jagoda A, Concussion guidelines step 1: systematic review of prevalent indicators. Neurosurgery. 2014;75(suppl 1):S3-S15. doi:10.1227/NEU.0000000000000433 25006974

[15] Ljungqvist J, Zetterberg H, Mitsis M, Blennow K, Skoglund T Serum neurofilament light protein as a marker for diffuse axonal injury: results from a case series study. J Neurotrauma. 2017;34(5):1124-1127. doi:10.1089/neu.2016.4496 27539721

[16] Mandrekar JN Receiver operating characteristic curve in diagnostic test assessment. J Thorac Oncol. 2010;5(9):1315-1316. doi:10.1097/JTO.0b013e3181ec173d 20736804

[17] Iliff JJ, Chen MJ, Plog BA, Impairment of glymphatic pathway function promotes tau pathology after traumatic brain injury. J Neurosci. 2014;34(49):16180-16193. doi:10.1523/JNEUROSCI.3020-14.2014 25471560 PMC4252540

[18] Albayram O, Kondo A, Mannix R, Cis P-tau is induced in clinical and preclinical brain injury and contributes to post-injury sequelae. Nat Commun. 2017;8(1):1000. doi:10.1038/s41467-017-01068-4 29042562 PMC5645414

[19] Castellani RJ, Perry G Tau biology, tauopathy, traumatic brain injury, and diagnostic challenges. J Alzheimers Dis. 2019;67(2):447-467. doi:10.3233/JAD-180721 30584140 PMC6398540

[20] Gill J, Latour L, Diaz-Arrastia R, Glial fibrillary acidic protein elevations relate to neuroimaging abnormalities after mild TBI. Neurology. 2018;91(15):e1385-e1389. doi:10.1212/WNL.0000000000006321 30209234 PMC6177279

[21] Asken BM, Bauer RM, DeKosky ST, Concussion BASICS III: serum biomarker changes following sport-related concussion. Neurology. 2018;91(23):e2133-e2143. doi:10.1212/WNL.0000000000006617 30404786

[22] McCrea M, Huber DL, Gill JM, Blood biomarkers in acute sport-related concussion: findings from the NCAA-DoD CARE Consortium. Poster presented at: Military Health System Research Symposium. August 21, 2019. Kissimmee, Florida. Accessed July 28, 2020. https://www.mhsrs.net/wp-content/uploads/2019/07/MHSRS-2019-Poster-Session-2-Information.pdf

[23] Nylén K, Öst M, Csajbok LZ, Increased serum-GFAP in patients with severe traumatic brain injury is related to outcome. J Neurol Sci. 2006;240(1-2):85-91. doi:10.1016/j.jns.2005.09.007 16266720

[24] Posti JP, Takala RSK, Runtti H, The levels of glial fibrillary acidic protein and ubiquitin c-terminal hydrolase-L1 during the first week after a traumatic brain injury: correlations with clinical and imaging findings. Neurosurgery. 2016;79(3):456-464. doi:10.1227/NEU.0000000000001226 26963330

[25] Shahim P, Tegner Y, Gustafsson B, Neurochemical aftermath of repetitive mild traumatic brain injury. JAMA Neurol. 2016;73(11):1308-1315. doi:10.1001/jamaneurol.2016.2038 27654934

[26] Bernick C, Zetterberg H, Shan G, Banks S, Blennow K Longitudinal performance of plasma neurofilament light and tau in professional fighters: the Professional Fighters Brain Health Study. J Neurotrauma. 2018;35(20):2351-2356. doi:10.1089/neu.2017.5553 29609512

[27] Meeter LH, Dopper EG, Jiskoot LC, Neurofilament light chain: a biomarker for genetic frontotemporal dementia. Ann Clin Transl Neurol. 2016;3(8):623-636. doi:10.1002/acn3.325 27606344 PMC4999594

[28] Oliver JM, Jones MT, Kirk KM, Serum neurofilament light in American football athletes over the course of a season. J Neurotrauma. 2016;33(19):1784-1789. doi:10.1089/neu.2015.4295 26700106

[29] Pattinson CL, Shahim P, Taylor P, Elevated tau in military personnel relates to chronic symptoms following traumatic brain injury. J Head Trauma Rehabil. 2020;35(1):66-73. doi:10.1097/htr.0000000000000485 31033745 PMC6814502

[30] Motamedi V, Kanefsky R, Matsangas P, Elevated tau and interleukin-6 concentrations in adults with obstructive sleep apnea. Sleep Med. 2018;43:71-76. doi:10.1016/j.sleep.2017.11.1121 29482817

[31] Holth JK, Fritschi SK, Wang C, The sleep-wake cycle regulates brain interstitial fluid tau in mice and CSF tau in humans. Science. 2019;363(6429):880-884. doi:10.1126/science.aav2546 30679382 PMC6410369

