# Supplementary material for: Plasma Biomarker Concentrations Associated With Return to Sport Following Sport-Related Concussion in Collegiate Athletes—A Concussion Assessment, Research, and Education (CARE) Consortium Study
Source: JAMA Netw Open. 2020 Aug 27;3(8):e2013191. doi: 10.1001/jamanetworkopen.2020.13191 (PMC7453307; doi:10.1001/jamanetworkopen.2020.13191)
Supplement: Supplement. — eTable. Demographic Characteristics of Participants With and Without Missing Biomarker Data at the Postinjury Period [file jamanetwopen-3-e2013191-s001.pdf]

# Supplementary Online Content

Pattinson CL, Meier TB, Guedes VA, et al; CARE Consortium Investigators. Plasma biomarker concentrations associated with return to sport following sport-related concussion in collegiate athletes—a Concussion Assessment, Research, and Education (CARE) Consortium study. *JAMA Netw Open*. 2020;3(8):e2013191. doi:10.1001/jamanetworkopen.2020.13191

**eTable.** Demographic Characteristics of Participants With and Without Missing Biomarker Data at the Postinjury Period

This supplementary material has been provided by the authors to give readers additional information about their work.

© 2020 Pattinson CL et al. *JAMA Network Open.*

**eTable. Demographic Characteristics of Participants With and Without Missing Biomarker Data at the Postinjury Period**

| mean ± SD or n (%) | **Completed post-injury biomarkers (n = 139)** | **No post-injury biomarkers (n = 169)** | **t/$\chi^2$** | ***P*** |
|---|---|---|---|---|
| Cohort | | | FET | .345 |
| University | 55 (48.7) | 58 (51.3) | | |
| Military Academy | 84 (43.1) | 111 (56.9) | | |
| Age | 18.9 ± 1.3 | 19.1 ± 1.3 | -1.35 | .179 |
| Sex (male) | 109 (78.4) | 122 (72.2) | FET | .130 |
| Years of Sport Participation | 10.1 ± 4.1 | 10.4 ±4.5 | -0.41 | .681 |
| Race | | | 7.17 | .208 |
| White | 86 (61.9) | 116 (68.6) | | |
| African American | 30 (21.6) | 31 (18.3) | | |
| Asian | 8 (5.8) | 2 (1.2) | | |
| Hawaiian or Pacific Islander | 2 (1.4) | 2 (1.2) | | |
| Multiple | 12 (8.6) | 14 (8.3) | | |
| Unknown/Not Reported | 1 (0.7) | 4 (2.4) | | |
| Ethnicity | | | 0.04 | .978 |
| Non-Hispanic | 118 (84.9) | 142 (84.0) | | |
| Hispanic | 7 (5.0) | 9 (5.3) | | |
| Unknown/Not Reported | 14 (10.1) | 18 (10.7) | | |
| ADHD+ | 10 (7.2) | 9 (5.3) | FET | .635 |
| SPORT | | | 15.06 | .089 |
| Football | 56 (52.3) | 51 (37.8) | | |
| Ice Hockey | 4 (3.7) | 16 (11.9) | | |
| Lacrosse | 6 (5.6) | 13 (9.6) | | |
| Rugby | 9 (8.4) | 14 (10.4) | | |
| Soccer | 19 (17.8) | 29 (21.5) | | |
| Other | 13 (12.1) | 12 (8.9) | | |
| Number of Prior Concussions | | | 4.04 | .401 |
| 0 | 79 (57.7) | 105 (62.9) | | |
| 1 | 46 (33.6) | 43 (25.7) | | |
| 2 | 10 (7.3) | 12 (7.2) | | |
| 3+ | 2 (1.5) | 7 (4.2) | | |

Note: ADHD; Attention Deficit Hyperactivity Disorder, FET; Fishers Exact Test (two-tailed)

© 2020 Pattinson CL et al. *JAMA Network Open.*
